# InGSA: integrating generalized self-attention in CNN for Alzheimer's disease classification

**DOI:** 10.3389/frai.2025.1540646

**Published:** 2025-03-12

**Authors:** Faisal Binzagr, Anas W. Abulfaraj

**Affiliations:** ^1^Department of Computer Science, King Abdulaziz University, Rabigh, Saudi Arabia; ^2^Department of Information Systems, King Abdulaziz University, Rabigh, Saudi Arabia

**Keywords:** Alzheimer's disease classification, generalized self-attention, CNN, transfer learning, transformer

## Abstract

Alzheimer's disease (AD) is an incurable neurodegenerative disorder that slowly impair the mental abilities. Early diagnosis, nevertheless, can greatly reduce the symptoms that are associated with the condition. Earlier techniques of diagnosing the AD from the MRI scans have been adopted by traditional machine learning technologies. However, such traditional methods involve depending on feature extraction that is usually complex, time-consuming, and requiring substantial effort from the medical personnel. Furthermore, these methods are usually not very specific as far as diagnosis is concerned. In general, traditional convolutional neural network (CNN) architectures have a problem with identifying AD. To this end, the developed framework consists of a new contrast enhancement approach, named haze-reduced local-global (HRLG). For multiclass AD classification, we introduce a global CNN-transformer model InGSA. The proposed InGSA is based on the InceptionV3 model which is pre-trained, and it encompasses an additional generalized self-attention (GSA) block at top of the network. This GSA module is capable of capturing the interaction not only in terms of the spatial relations within the feature space but also over the channel dimension it is capable of picking up fine detailing of the AD information while suppressing the noise. Furthermore, several GSA heads are used to exploit other dependency structures of global features as well. Our evaluation of InGSA on a two benchmark dataset, using various pre-trained networks, demonstrates the GSA's superior performance.

## 1 Introduction

Alzheimer's disease (AD) is a type of dementia that is not curable, which becomes worse over years as it affects the human brain, but early diagnosis helps to minimize the symptoms and the management of the patient (McKhann et al., 1984). Its manifestation involves impaired memory because patients cannot organize or recall information properly, and poor judgment that renders the affected persons completely helpless and in need of care as the disease develops (Choi et al., 2020). The probability raised from 2% at 65 years to 35% at 85 years for AD. Approximately 26.6 million people had it in 2006; the figure rose to over 55 million in 2020 and is expected to reach 152 million by 2050 (Gunawardena et al., 2017). Neuronal loss and synaptic impairment can occur at least one or two decades before disease onset (Böhle et al., 2019). It is essential to detect AD in the prodromal stage, which is characterized by moderate cognitive impairment (MCI), as there is currently no cure. Early MCI (EMCI) is a cognitive impairment stage that precedes MCI (Kang et al., 2020). The early detection of EMCI has the potential to prevent the progression of EMCI to AD. The importance of diagnosing MCI patients has been emphasized by studies that has identified the distinctions between early MCI (EMCI) and late MCI (LMCI) groups (Nozadi et al., 2018; Edmonds et al., 2019; Zhang T. et al., 2019). MCI has a symptom profile that is similar, but less severe, to AD (Varatharajah et al., 2019). Nowadays, this disease is also defined as mild cognitive impairment associated with the existence of Alzheimer's disease; according to recent investigations, ~80% of patients diagnosed with MCI develop AD in 7 years. For monitoring of variations in the densities of the brain tissues, magnetic resonance imaging (MRI) and positron emission tomography (PET) are frequently used since they do not include the invasion of the tissues (Ramzan et al., 2020; Gao, 2021). Neuroimaging, especially using MRI, is crucial for the study of the nervous system structures more closely (Tuvshinjargal and Hwang, 2022); this test helps in diagnosis of certain diseases such as tumors and cancer (Tehsin et al., 2024). MRI does work in the case of Alzheimer's; it allows capturing structural changes in the brain, for instance, the reduction of certain regions and the appearance of new formations, heterogeneous density, and the presence of abnormal substances typical of the disease (Simic et al., 2009).

In recent years, medical imagery such as MRI has been used with machine learning (ML) and deep learning (DL). These methods are used in health checks and early AD diagnosis. They also excel at categorizing images in health and computer vision (Nasir et al., 2021, 2020, 2022; Yousafzai et al., 2024; Nasir et al., 2023). In recent decades, neuroimaging data have grown, allowing ML and DL algorithms to better characterize AD. The authors used such methodologies to offer prospective AD diagnosis and prognostic outcomes (Nagarajan et al., 2021). These works executed features from several image processing pipeline streams using random forest classifier, decision tree, or support vector machine (SVM). Lately, DL techniques have showed potential in medical imaging with good picture classification accuracy (Ajagbe et al., 2021). Automatic feature extraction from images using CNNs and transfer learning (TL) is more efficient than typical ML methods (Raju et al., 2021). However, working with medical data is problematic due to imbalanced dataset, including AD. In this strategy, various sample sizes are used for different classes, the model is always biased, and it cannot generalize beyond the training dataset. DL models can process raw data better than simple feed forward, but they can overfit when solving complicated problems such as class imbalance. In real-world circumstances, such models perform poorly in generalization, efficacy, and reliability. The main contribution of this study is as follows:

- We introduce a contrast enhancement method called haze-reduced local-global, inspired by the haze reduction principle.
- We suggest a new global CNN-transformer architecture, InGSA, for the classification of multiclass AD. A pre-trained CNN is integrated with a specialized transformer module in InGSA network.
- This network, comprised of several generalized self-attention module (GSA), is designed to effectively capture extensive feature dependencies across different brain regions by establishing global connections along both the channel and spatial dimensions.
- The InGSA model is tested on a two publicly available dataset, where we also use various pre-trained CNN models to demonstrate its effectiveness. Furthermore, we perform a comparative analysis between InGSA and modern attention mechanisms, as well as the latest approaches in multiclass AD classification.

The structure of this research is comprehensively examined in the following manner: Related works are detailed in Section 2. Section 3 delineates the fundamental concepts and proposed methodology. The experimental results are the subject of Section 4. The study is concluded in Section 5.

## 2 Related work

Over the past few years, the usage of DL methods for the identification of AD has received much attention (Mohammed et al., 2021; Ahmed et al., 2022; Menagadevi et al., 2023). For instance, a study employed DL with stacked auto-encoders and uses the softmax function in the final layer to address problem of bottlenecks. Their approach needed far less training data compared to their peers, as well as very small input to classify several groups with ~87.70% accuracy. One of the observations from the current study was that the use of several features improves classification (Frizzell et al., 2022). Furthermore, a classification framework was built, based on the use of multiple different input databases since it is complementary. To combine features from different modalities, they used a process known as non-linear graph mixture model. Using this method, the areas under the curve were calculated with 98.1% accuracy when differentiating between AD and CN images, 82.40% between NC and MCI images, with the overall classification performance being 77.90% (Guo and Zhang, 2020).

A novel rapid, low-cost, and efficient diagnostic model was implemented using brain MRI scans. They used DenseNet121 model which is a computationally heavy model, and to this model, they achieved an accuracy of 87% in detecting the disease. To rectify this, the authors employed an idea of fine-tuning two models of AlexNet and LeNet models where features were extracted in three ways through parallel filters. The new model they came up with was able to predict the disease with an accuracy of 93% (Hazarika et al., 2023). In the same manner, the researchers in Acharya et al. (2021) used VGG-16 based CNN transfer learning to diagnose AD with an overall accuracy of 95.7%. Another study used DL for distinguishing dementia and Alzheimer's from the MRI images (Murugan et al., 2021).

The approach used in Murugan et al. (2021) learns individual Alzheimer's likelihood using multilayer perceptron representations and also generates disease probability heat maps from brain region activity. To overcome the problem of class imbalance, the samples are divided in equal proportion. The five ADNI subtypes consist of 1,296 images comprising of AD, MCI, EMCI, LMCI, and CN images processing the DEMNET model by resizing the images to 176 × 176 and obtained an accuracy of 84.83%. In the same way, Oktavian et al. (2022) presented the fine-tuned ResNet18 model for distinguishing between MCI, AD, and CN using MRI and PET datasets. This model incorporated transfer learning and used the technologies such as weighted loss function for ascending the class imbalance, and mish activation function to augment its accuracy, and it obtained 88.3% overall classification. On the other hand, the authors in Dyrba et al. (2021) adopted a CNN with 663 T1-weighted MRI scans belonging to dementia and amnestic MCI patients. To confirm their model, they performed cross-validation and used an additional three datasets that included an overall of 1,655 cases. To further provide the clinical relevance of the method, they correlated the relevance scores to the hippocampal volume. A friendly model assessment tool was created through importance maps of 3D CNN, achieving accuracy of 94.9% of AD vs. CN. A particular drawback of many papers on the detection of Alzheimer's is related to the imbalance of classes, which creates problems of overfitting and lowering predictive ability in almost all existing deep learning models. The yield is further magnified by the fact that realistic training data are also scarce. To overcome this, we utilized the data augmentation approach to balance datasets and improve DL results since the technique synthesizes new data samples.

## 3 Proposed methodology

**Figure 1:**
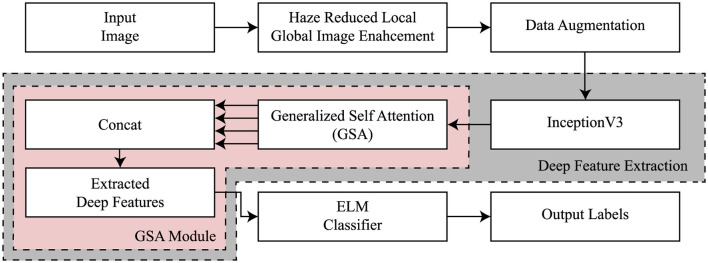
General flow of the proposed framework.

The configuration of the proposed InGSA is illustrated in Figure 1, comprising a fine-tuned CNN model, a generalized self-attention (GSA), and a classifier. The fine-tuned CNN models aid in extracting abstract feature representations from the input MR images. The GSA block has various components to comprehend global interdependence across spatial and channel dimensions, facilitating the extraction of more nuanced and category-specific information. The extreme learning machine (ELM) classifier is employed to categorize AD. This section offers a comprehensive overview of the InGSA architecture and its fundamental components.

### 3.1 Haze reduced local global image enhancement

Traditional haze elimination procedures are developed for improving the visual distinctiveness of scenes by increasing the contrast and color saturation. By applying these techniques, the total clarity of the scene which is captured in the given image is likely to be enhanced. In this research, we formally propose a new type of contrast enhancement method that adopts both haze removal and local-global transformation techniques.

Let *D* denotes a complex image database that is composed of *N* images. While the original image is represented by the dimensions of *N* × *M* × 3 as *I*(*x, y*), the *Y*(*x, y*) denotes the improved image. First, a haze reduction method utilizing the dark channel prior is employed on the first image. This process of haze reduction can be mathematically expressed as follows:


(1) $$\begin{array}{l} C\left(x\right)=\gamma \left(x\right)j\left(x\right)+l\left(1-t\left(x\right)\right) \end{array}$$

where *C* denotes the measured intensity values, γ represents the scene radiance, *j*(*x*) designates the transmission map, and *l* denotes the atmospheric light intensity. The dehazing algorithm utilized aims to restore the scene radiance γ based on the estimations of both the transmission map and the atmospheric light, as expressed in the following manner:


(2) $$\begin{array}{l} \gamma \left(x\right)=\frac{C\left(x\right)-\alpha }{\max\left(t\left(x\right),t_{0}\right)}+\alpha \end{array}$$

The resulting γ(*x*) is subsequently employed to calculate the global contrast of an image using the following equation:


(3) $$\begin{array}{l} G_{0}=\left(1+g_{k}\right)\times \left(G_{i}-k_{\text{mean}}\right)+\sigma \end{array}$$

In this regard, *G*_0_ stands for the global contrast image of the original image while *g*_*k*_ represents gain factor of global contrast, *G*_*i*_ for the value of pixel γ(*x*), *k*_mean_ for the overall average pixel value of γ(*x*), and σ for the standard deviation of γ(*x*). In the subsequent step, we assessed the local contrast of the haze-reduced image using the following mathematical expression:


(4) $$\begin{array}{l} H\left(x,y\right)=\frac{LC}{\sigma \left(i,j\right)+\varphi }\times \mu \left(x,y\right) \end{array}$$

where *LC* for local contrast, φ for a small constant, and μ(*x, y*) means the mean value of the dehazed image. Finally, these two resultant images of local and global contrast were incorporated toward a single image in this way that we adopt the following mathematical formula to produce the final enhancement output.


(5) $$\begin{array}{l} Y(x,y)=[G(x,y)+H(x,y)]-I(x,y)] \end{array}$$

### 3.2 Deep transfer learning

**Figure 2:**
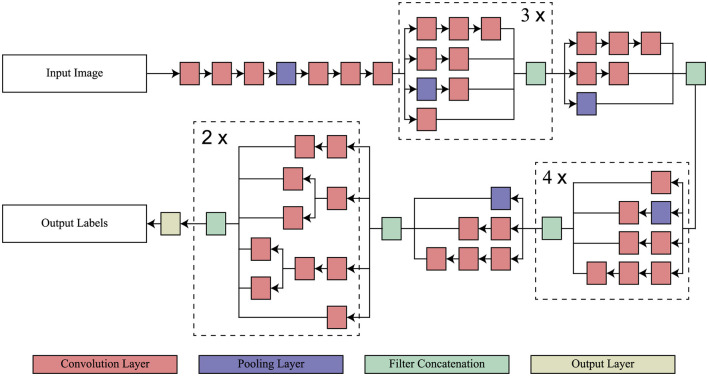
Architecture of InceptionV3 model.

InceptionV3 is a directed acyclic graph (DAG) network that has 316 layers and 350 links that include 94 as convolutional layers (Szegedy et al., 2016). Such a structure facilitates the provision of adequate employment of complicated dependency relations in the network, having many inputs and outputs at different layers. Differently from the standard CNN model in which the filter size is fixed throughout the layers of that model, InceptionV3 has different filter sizes within the same layer, which increases its capability of feature extraction on the data. Originally trained on ImageNet (Deng et al., 2009) on which includes over one million images split into one thousand categories, the InceptionV3 has the ability to read most features. The model takes images of input size 299 × 299 × 3. In this study, the model has been adapted to classify various stages of AD for which transfer learning from the ImageNet training phase has been applied to achieve efficient medical image classification. The architecture of InceptionV3 model is shown in Figure 2.

Transfer learning (Pan and Yang, 2009) is a popular method in recognition and detection tasks, allowing for improved model performance by leveraging pre-trained models. In this context, the domain D consists of a feature vector *Y* = *y*_1_, *y*_2_, ⋯, *y*_*n*_ with a corresponding probabilistic distribution *P*(*Y*), forming *B* = *Y, P*(*Y*). The task, denoted as *T*, consists of the ground truth *Z* = *z*_1_, *z*_2_, ⋯, *z*_*n*_. The function can be expressed in probabilistic form as *P*(*z*|*y*). In the context of transfer learning, this can be represented concerning the source domain as *B*_*T*_ = (*x*(*T*_1_), *x*(*T*_2_)), (*x*(*T*_2_), *x*(*T*_2_)), ⋯, (*x*(*T*_*n*_), *x*(*T*_*n*_)) along with the learning rate *S*_*T*_. The target output is denoted as *B*_*S*_ = (*x*(*S*_1_), *x*(*S*_2_)), (*x*(*S*_2_), *x*(*S*_2_))⋯, (*x*(*S*_*n*_), *x*(*S*_*n*_)), and the associated function for the targeted neural network is represented as *S*_*S*_. The primary objective of transfer learning is to improve the learning rate for predicting the target object by utilizing the recognition function *F*_*S*_(.), which is informed by training on both *B*_*T*_ and *B*_*S*_, where *B*_*T*_ ≠ *B*_*S*_ and *S*_*T*_ ≠ *S*_*S*_. Inductive transfer learning proves to be effective in pattern recognition tasks. An annotated dataset is essential for efficient training and evaluation when implementing inductive transfer learning. This process can involve distinct class labels *Z*_*T*_ ≠ *Z*_*S*_ and differing distributions *P*(*Z*_*T*_|*Y*_*T*_) ≠ (*Z*_*S*_|*Y*_*S*_).

### 3.3 Generalized self-attention module

**Figure 3:**
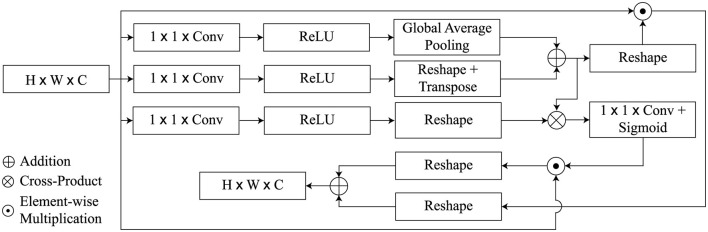
Overview of the GSA block.

The proposed GSA module is aimed at achieving detailed description of AD characteristics while avoiding irrelevant features. Its architecture was influenced by the self-attention mechanisms employed in GCNet (Cao et al., 2019; Zhang et al., 2019), as illustrated in Figure 3. However, unlike these methods, it positions global dependency across both spatial and channel dimensions at the same time. Spatial attention worked for the relationships of the global features in the spatial location, while channel attention worked on the importance of a point channel out of all the channels.

As initial input for the GSA module, we utilize the high level activation maps *Z* ∈ ℝ^*H* × *W* × *C*^, whereas the GSA module returns the refined feature maps $Z_{gs}\in {\mathbb{R}}^{H\times W\times C}$. The feature map is divided into the keys, queries, and values, similar to a transformer architecture, which is supported by three attributes *Q*, *K*, and *V*.

The query function *q*(*Z*) is defined by a convolution of 1 × 1 consisting of *C*′ = *C*/8 channels and global average pooling to attain the vector *Q*(*Z*) ∈ ℝ^1 × *C*′^. On the other hand, the key and value functions are carried out by 1 × 1 convolution followed by reshape operations but without global average pooling and the outputs are maps *K*(*Z*) ∈ ℝ^*HW* × *C*′^ and *V*(*Z*) ∈ ℝ^*HW* × *C*′^). Next, The spatial attention weights are generated by calculating the matrix product between *Q* and *K* and applying a softmax activation function given as


(6) $$\begin{array}{l} Z^{\prime }=\phi \left(q\left(Z\right)⊗k{\left(Z\right)}^{T}\right) \end{array}$$

With regard to the abbreviations used here, we have ⊗ indicating the cross-product of the matrix, ϕ which stands for the softmax activation function of the formula and the double dagger *T* showing the operation of matrix transposition. Following this, the spatial attention feature map $Z_{sp}\in {\mathbb{R}}^{H\times W\times C}$ derived by performing element-wise multiplication among *Z*′ and *Z* is as follows:


(7) $$\begin{array}{l} Z_{sp}=\text{reshape}\left(Z^{\prime }\right)⊙Z \end{array}$$

Similarly, a matrix cross-product of *Z*′ with *v*(*Z*) leads to the channel attention weights which are passed through a 1 × 1 convolution layer and a sigmoid non-linearity. It also increases the channels from *C*′ to *C*. This process is also called linear embedding. The mathematical formulation for this global transformation is given by


(8) $$\begin{array}{l} Z^{\prime \prime }=\sigma \left(\text{conv}\left(Z^{\prime }⊗v\left(Z\right)\right)\right) \end{array}$$

Next, the channel-wise attention maps $Z_{ch}\in {\mathbb{R}}^{H\times W\times C}$ are calculated as


(9) $$\begin{array}{l} Z_{ch}=Z^{\prime \prime }⊙Z \end{array}$$

Finally, we integrate the spatial attention feature maps *Z*_*sp*_ and the channel attention maps *Z*_*ch*_ by taking their weighted sum, producing the refined attention feature map $Z_{gs}\in {\mathbb{R}}^{H\times W\times C}$, defined as


(10) $$\begin{array}{l} Z_{gs}=W_{1}Z_{sp}+W_{2}Z_{ch} \end{array}$$

where *W*_1_ and *W*_2_ are two trainable scalar weights. In summary, GSA obtains the channel-wise and spatial dependencies concurrently from MR images and then improves the features representation. We merge the feature attention maps produced by the GSA heads through concatenation, preceding 1 × 1 convolution to generate the final output of the proposed GSA, denoted as $Z_{tm}\in {\mathbb{R}}^{H\times W\times C}$. Mathematically, *Z*_*t*_*m* can be represented as follows:


(11) $$\begin{array}{l} Z_{tm}=\text{concat}\left(Z_{gs}^{1},Z_{gs}^{2},\ldots ,Z_{gs}^{h}\right) \end{array}$$

In this study, *h*, representing the number of GSA heads, is set empirically to a value of 4. This specific choice of *h* = 4 was determined empirically.

### 3.4 Classification

The ELM [42] was used as a classifier to differentiate AD stages. Given *z* sample (*Z, o*), the ELM's output with no errors can be mathematically expressed as follows:


(12) $$\begin{array}{l} o=\sqrt{\sum \alpha t\left(w_{i}+b\right)} \end{array}$$

In this instance, the activation function is denoted by *t*(·), and the input and output samples are *Z* and *o*, respectively. The variables *w* and *b* are weights and bias, respectively, and α is the weight coefficient. The output O is provided as *O* = *Hα* whereby *O* = (*o*_1_, *o*_2_, ⋯, *o*_*n*_) symbolizes the output vector and α = (α_1_, α_2_, …, α_*m*_) denotes the weight vector. The hidden layers can be expressed as


(13) $$\begin{array}{l} H=\left[\begin{array}{ccc} t\left(w_{1}i_{1}+b_{1}\right) & \cdots & t\left(w_{n}i_{1}+b_{n}\right)\\ \vdots & \ddots & \vdots \\ t\left(w_{1}i_{m}+b_{1}\right) & \cdots & t\left(w_{n}i_{m}+b_{n}\right) \end{array}\right] \end{array}$$

The number of nodes in the hidden layer needs to be below the total amount of samples. Description of the structured model of a single hidden-layer ELM neural network utilized for AD classification is provided in Equation 13. The hidden layer, denoted by *H*, is composed of nodes and activation functions *t*(·). Weights *w*_*i*_ and biases *b* are connected to each hidden layer node, with *i* ranging from 1 to *m* and representing input variables. The formula used in the production of the output of the hidden layer *O* is the summation of the product between each of the node's activation function and weights then passed through *t*(·). The mechanism can be mathematically represented as *O* = *Hα*. Equation 13 defines the structure of the hidden layer, which further elucidates that the *H* is a concatenation of *n* nodes. The weighted sum of input features *i* = (*i*_1_, *i*_2_, ⋯, *i*_*m*_) is computed for each node's activation by utilizing weights *w*_*i*_ and biases *b* for each node. The function *t*(·) adds non-linearity and provides the network with ability to learn more complex input data patterns. Determining the quantity of nodes in the hidden layers is essential; they should be fewer than the amount of samples to avert overfitting. During training, we obtain the weights and biases to minimize the mapping function between the input features and the associated output for AD classification.

## 4 Experimental results

The analysis and experimental results of the proposed models are detailed in this section. The presentation includes information regarding the dataset, implementation characteristics, and comparison analysis.

### 4.1 Experimental setup and dataset

The model was trained on a high-performance machine equipped with an Intel Core i9-14900HX processor and an NVIDIA RTX 4090 GPU, providing substantial computational power for deep learning tasks. The system included 64GB of DDR5 RAM operating at 5,600MT/s, ensuring efficient handling of large-scale data. CUDA 12.6 was utilized to enable GPU-accelerated training. The model was trained with a learning rate of 0.0001, a value chosen to balance the stability and convergence speed of the training process.

In this experiment, ADNI dataset was used which consisted of five classes: AD, CN, EMCI, LMCI, and MCI. The original number of images varied significantly across classes, with AD, CN, and MCI having thousands of images, while EMCI and LMCI had considerably fewer as shown in Table 1. To address this class imbalance, data augmentation was applied exclusively to the LMCI class, which originally had only 144 images. Through augmentation, the LMCI class was expanded to 432 images, increasing the total number of samples used in the training process. For the other classes, 500 images were randomly selected from AD and CN, while all available images were used for EMCI and MCI.

**Table 1:** ADNI dataset image count before and after augmentation.

**Classes**	**No. of images**	**Augmented**	**No. of utilized images**
AD	8,346	n/a	500
CN	8,650	n/a	500
EMCI	480	n/a	480
LMCI	144	432	432
MCI	1,155	n/a	500

The augmentation methods used to enhance the LMCI class included rotation, scaling, and flipping. Rotation involved rotating images by various angles to introduce diversity without altering key signal features. Scaling was applied to adjust the size of images while maintaining their aspect ratio, simulating variability in data capture. Flipping, both horizontally and vertically, was also used to further diversify the dataset, making the model more robust to orientation changes. These augmentation techniques were critical in improving class balance and ensuring better generalization during model training.

Another dataset used in this experiment is the Open Access Series of Imaging Studies (OASIS), a widely utilized resource for neuroimaging research, particularly in the study of brain health and dementia. Table 2 presents the distribution of images from the OASIS dataset across four classes: Mild Dementia, Moderate Dementia, Non-Demented, and Very Mild Dementia.

**Table 2:** Total number of images and number of images utilized from OASIS dataset.

**Classes**	**No. of images**	**No. of utilized images**
Mild dementia	5,002	500
Moderate dementia	488	488
Non-demented	67,200	500
Very mild dementia	13,700	500

### 4.2 ADNI results

**Figure 4:**
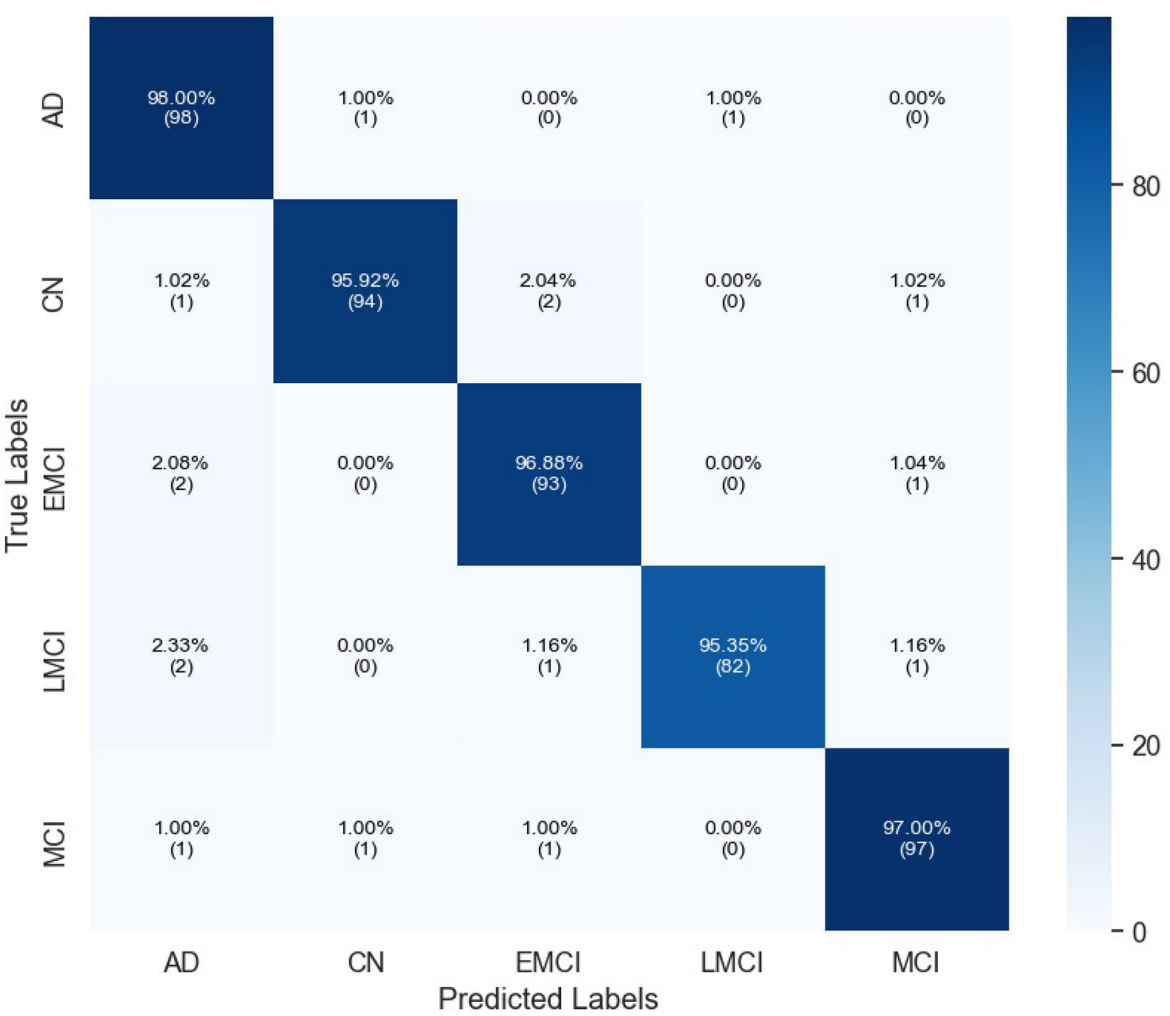
Confusion matrix of proposed InGSA on ADNI Dataset.

Table 3 indicates the performances of the proposed model in terms of classification for Alzheimer's detection under the different cognitive conditions. The network can accurately predict an image with an average of 96.67% and high values of precision, recall, and F1-scores for all classes. For the LMCI class, the study realized a precision of 98.80%, while at the same time, AD has the highest recall of 98.00% to show effective detection. All classes have an F1-score of more than 96%, and it can be seen that the approach balanced precision and recall. The AUC values are also high, and AD reached 98.21%. Confusion matrix for proposed model is given in Figure 4.

**Table 3:** Classification performance of InGSA on ADNI dataset.

**Class**	**Precision (%)**	**Recall (%)**	**F1-score (%)**	**AUC (%)**
AD	94.23	98.00	96.08	98.21
CN	97.92	95.92	96.91	97.70
EMCI	95.88	96.88	96.37	97.92
LMCI	98.80	95.35	97.04	97.55
MCI	97.00	97.00	97.00	98.11
Accuracy	96.67			
Macro average	97.76	96.63	96.68	97.90
Weighted average	96.71	96.67	96.67	97.91

**Figure 5:**
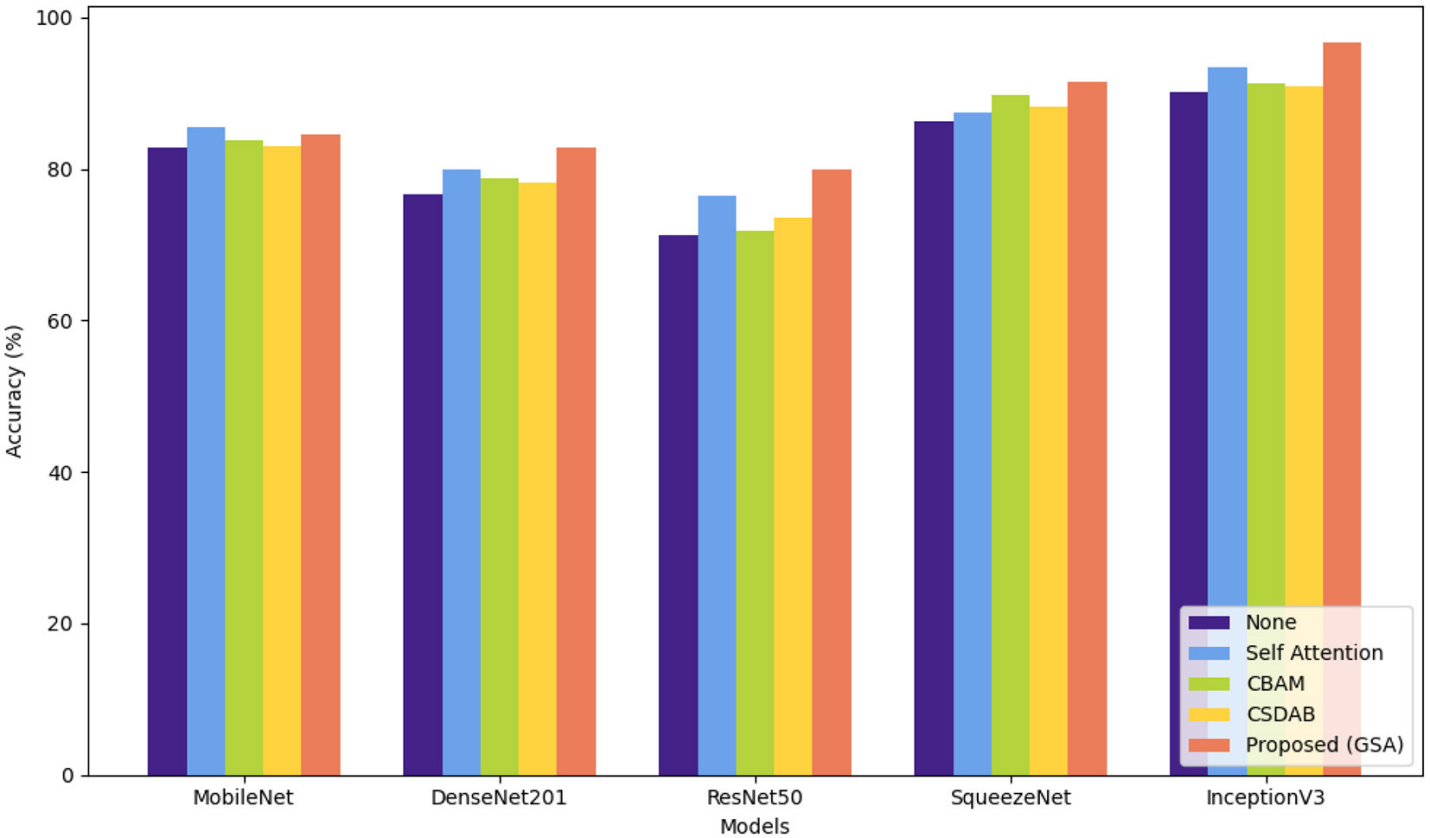
Visual comparison of various attention mechanisms on ADNI dataset.

Table 4 gives a quantitative analysis of the number of correct detections of the various models augmented with different attention mechanisms: MobileNet (Mob), DenseNet201 (Den), ResNet50 (Res), SqueezeNet (Sq), and InceptionV3 (InV3). With the proposed GSA, the largest performance improvements were achieved with InceptionV3, from 90.17% to 96.67% (with attention) and with SqueezeNet from 86.32% to 91.43%. Here, DenseNet201 shows improvement of 6.22% from 76.65 to 82.87, while ResNet50 goes from 71.27% to 79.98%. Self-Attention (Zhang et al., 2019) also presented substantial enhancements for InceptionV3 from 93.15% to 93.44%, as well as for SqueezeNet from 86.59% to 87.36%. Both convolutional block attention module (CBAM) (Woo et al., 2018) and channel split dual attention block (CSDAB) (Dutta and Nayak, 2022) result in moderate accuracy increases, with CBAM improving SqueezeNet to 89.78% and CSDAB raising it to 88.24%. In general, GSA consistently improves accuracy in all models being tested. Visual analysis of attention mechanisms with pre-trained model on ADNI dataset is shown in Figure 5.

**Table 4:** Performance comparison of different attention mechanisms.

**Attention mechanism**	**Accuracy (%)**				
	**Mob**	**Den**	**Res**	**Sq**	**InV3**
None	82.76	76.65	71.27	86.32	90.17
Self attention	85.43	79.89	76.39	87.36	93.44
CBAM	83.70	78.72	71.89	89.78	91.23
CSDAB	83.00	78.21	73.47	88.24	90.87
Proposed (GSA)	84.54	82.87	79.98	91.43	96.67

Table 5 shows accuracy and F1-score when comparing the current existing models, namely, vision transformer (ViT), data efficient image transformer (DEiT), and pyramid vision transformer (PVT) with the InGSA model proposed in this study. Out of all the models, the DEiT comes with the highest accuracy and F1-score with accuracies of 89.44%, and F1-score of 88.12%, with PVT coming second with accuracies of 88.48% and F1-scores of 85.98%. ViT has the worst performance with an accuracy of 86.24% and the F1-score at 84.67%. Comparing the proposed model InGSA with others, it is clear that the proposed model InGSA outperforms the other models with accuracy of 96.67% and F1-score of 96.68% that indicate effectiveness of the proposed model InGSA.

**Table 5:** Comparison of proposed InGSA with transformer-based models on ADNI dataset.

**Model**	**Accuracy (%)**	**F1-score (%)**
ViT	86.24	84.67
DEiT	89.44	88.12
PVT	88.48	85.98
InGSA	96.67	96.68

### 4.3 OASIS results

**Figure 6:**
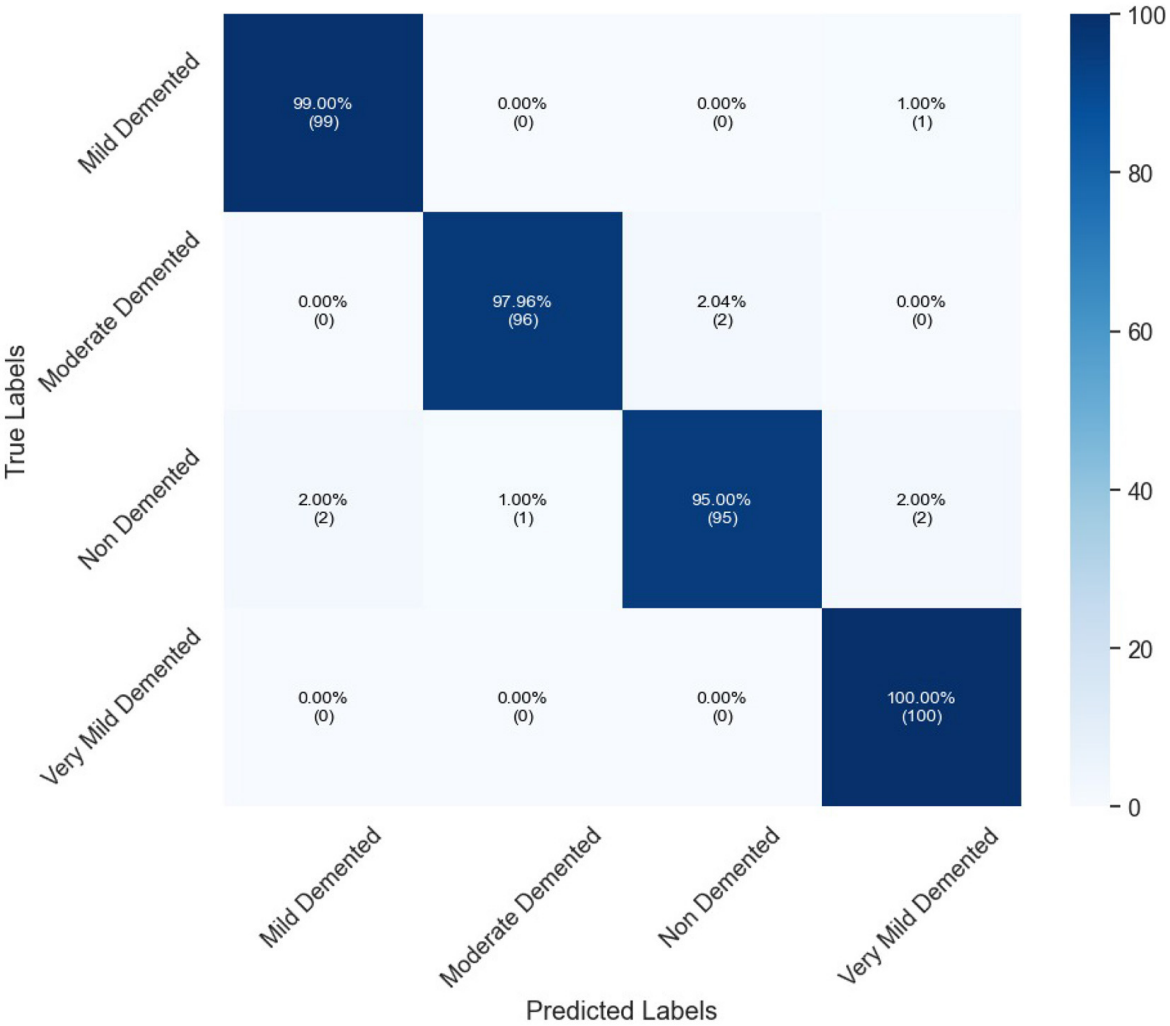
Confusion matrix of proposed InGSA on OASIS dataset.

Table 6 presents the classification performance for different categories of dementia using OASIS dataset. High precision of all classes set by the model, specifically Moderate Demented presenting 98.97% and Mild Demented 98.02%. The Recall is exceptional for Very Mild Demented at 100% which means that all the cases belonging to this class are identified rightly. The F1-score, therefore, averaged over all classes is unbiased, being 96.45% for Non-Demented and 98.52% for both Mild and Very Mild Demented classes. AUC's are high, notably Very Mild Demented with a highest of 99.50%. In general, the proposed model renders high performance in the presented study with the overall accuracy of 97.99%. Figure 6 depicts confusion matrix of proposed model for OASIS dataset.

**Table 6:** Classification performance of InGSA on OASIS dataset.

**Class**	**Precision (%)**	**Recall (%)**	**F1-score (%)**	**AUC (%)**
Mild demented	98.02	99.00	98.51	99.16
Moderate demented	98.97	97.96	98.46	98.81
Non-demented	97.94	95.00	96.45	97.16
Very mild demented	97.09	100.00	98.52	99.50
Accuracy	97.99			
Macro average	98.00	97.99	97.98	98.66
Weighted average	98.00	97.99	97.98	98.66

**Figure 7:**
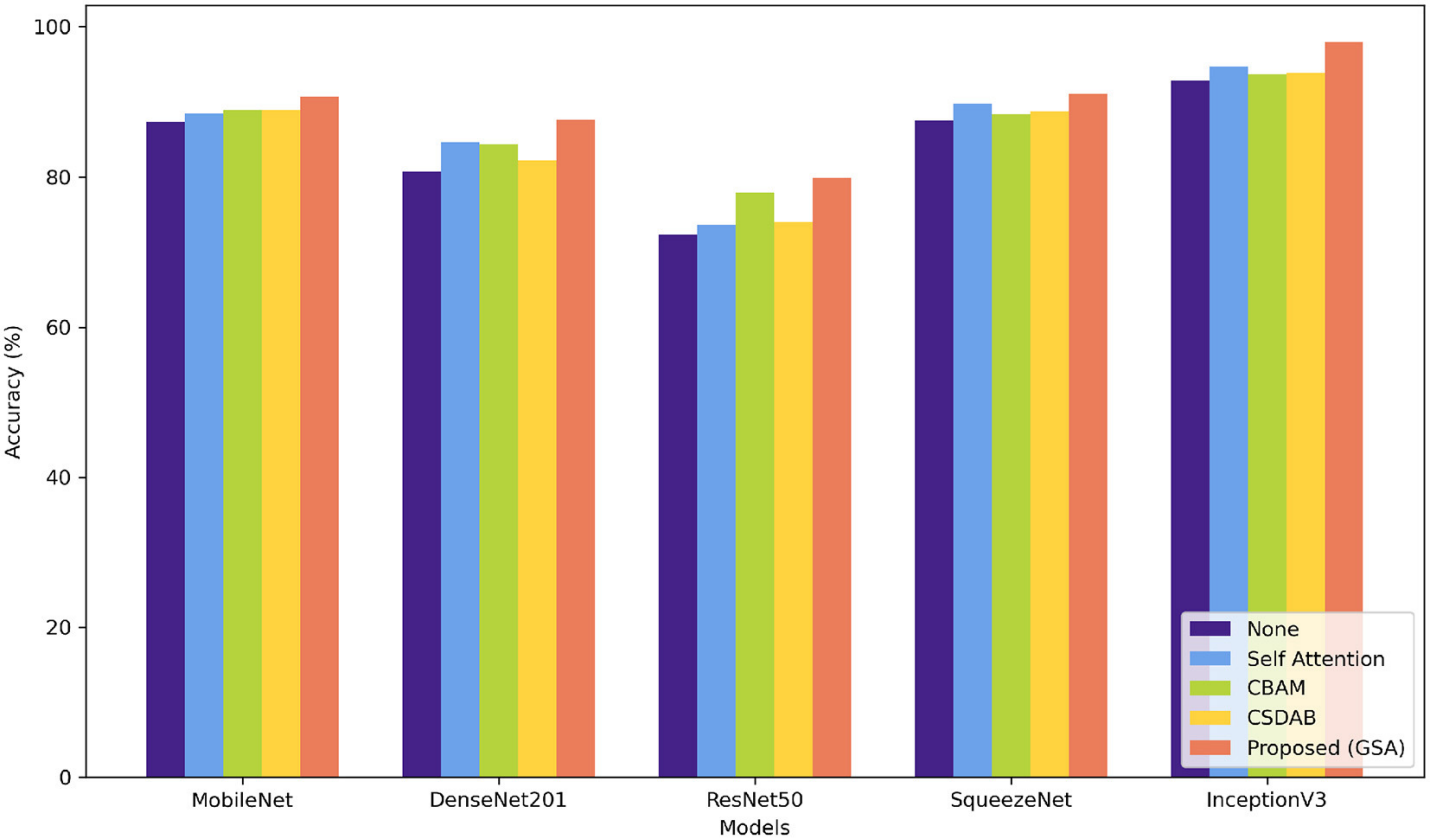
Visual comparison of various attention mechanisms on OASIS dataset.

Table 7 shows the percentage of classification accuracy of multiple models when using the OASIS dataset, with different attention mechanisms. All the attention approaches improve on the baseline accuracy of all the models including the proposed GSA model. For instance, they achieve 90.74% accuracy with MobileNet and an outstanding 97.99% with InceptionV3 confirming how efficient the proposed approach is in enhancing model accuracy. Self-attention mechanism also plays a useful role, especially in MobileNet and InceptionV3 models and in this experiment reached a throughput of 88.45 and 94.76%, correspondingly. While structure imported with CBAM and CSDAB mechanisms may be less fortunate than the GSA model, it has revealed improvements. Figure 7 illustrates the visual analysis of attention mechanisms using a pre-trained model on the OASIS dataset.

**Table 7:** Performance comparison of different attention mechanisms.

**Attention mechanism**	**Accuracy (%)**				
	**Mob**	**Den**	**Res**	**Sq**	**InV3**
None	87.35	80.78	72.34	87.54	92.87
Self attention	88.45	84.67	73.67	89.79	94.76
CBAM	88.89	84.32	77.93	88.38	93.62
CSDAB	88.95	82.19	74.05	88.75	93.90
Proposed (GSA)	90.74	87.64	79.85	91.02	97.99

The findings in Table 8 reveal that all algorithms provide high accuracy, where proposed InGSA model performs the best with accuracy of 97.99% and F1-score of 97.88%. This implies that InGSA is proud not only to classify instances accurately but also to have a low percentage of false positive and false negative. Next is the PVT model which gives classification accuracy of 93.79% and F1-score of 92.61% demonstrating the good classification prowess of the model. The performance of both ViT and DEiT models is reasonable, with accuracies of 91.65% and 89.43%, respectively.

**Table 8:** Comparison of proposed InGSA with transformer-based models on OASIS dataset.

**Model**	**Accuracy (%)**	**F1-score (%)**
ViT	91.65	86.78
DEiT	89.43	89.12
PVT	93.79	92.61
InGSA	97.99	97.88

## 5 Conclusion

Alzheimer's disease, diagnosed and classified with multiclass datasets in the early stage, needed a proficient automatic system identification. This study puts forward a CNN-Transformer model to diagnose Alzheimer's cases from multiclass datasets using transfer learning. First, a method of contrast enhancement is utilized to help better visualize important features. Furthermore, we introduce a new global CNN-transformer network known as InGSA for multiclass AD classification to facilitate end-to-end training. The InGSA architecture is based on the CNN and transformer, and GSA blocks are placed on top of pre-trained InceptionV3 model. GSA blocks are important for expression subscalar detection of global dependencies of features. The GSA component improves the extraction of detailed information by learning channel-wise and spatial-wise attention weights at the same time. In-depth experiments on two benchmark datasets demonstrate that our proposed InGSA achieves superior performance compared to the state-of-the-art techniques. Furthermore, GSA yields better results than other traditional attention methods. For the future works, we aim to test GSA on more extensive set and diverse dataset, and we also want to apply our proposed method in the other vision-related tasks.

## Data Availability

Publicly available datasets were analyzed in this study. This data can be found at: https://adni.loni.usc.edu/data-samples/adni-data/.
